# Supplementary material for: Spatially congruent sites of importance for global shark and ray biodiversity
Source: PLoS One. 2020 Jul 6;15(7):e0235559. doi: 10.1371/journal.pone.0235559 (PMC7337351; doi:10.1371/journal.pone.0235559)
Supplement: S1 Fig — General richness for (a) total species, (b) evolutionarily distinct (ED) species, and (c) endemic species. (d-f) Threatened subsets of richness patterns for (d) total species, (e) evolutionarily distinct (ED) species, and (f) endemic species. Geographic coordinate system is in NAD83, projected coordinate system is lambert equal area. The data used for this figure under CC BY license is granted permission from the International Union for the Conservation of Nature (IUCN), original copyright 2011. (DOCX) [file pone.0235559.s001.docx]

**
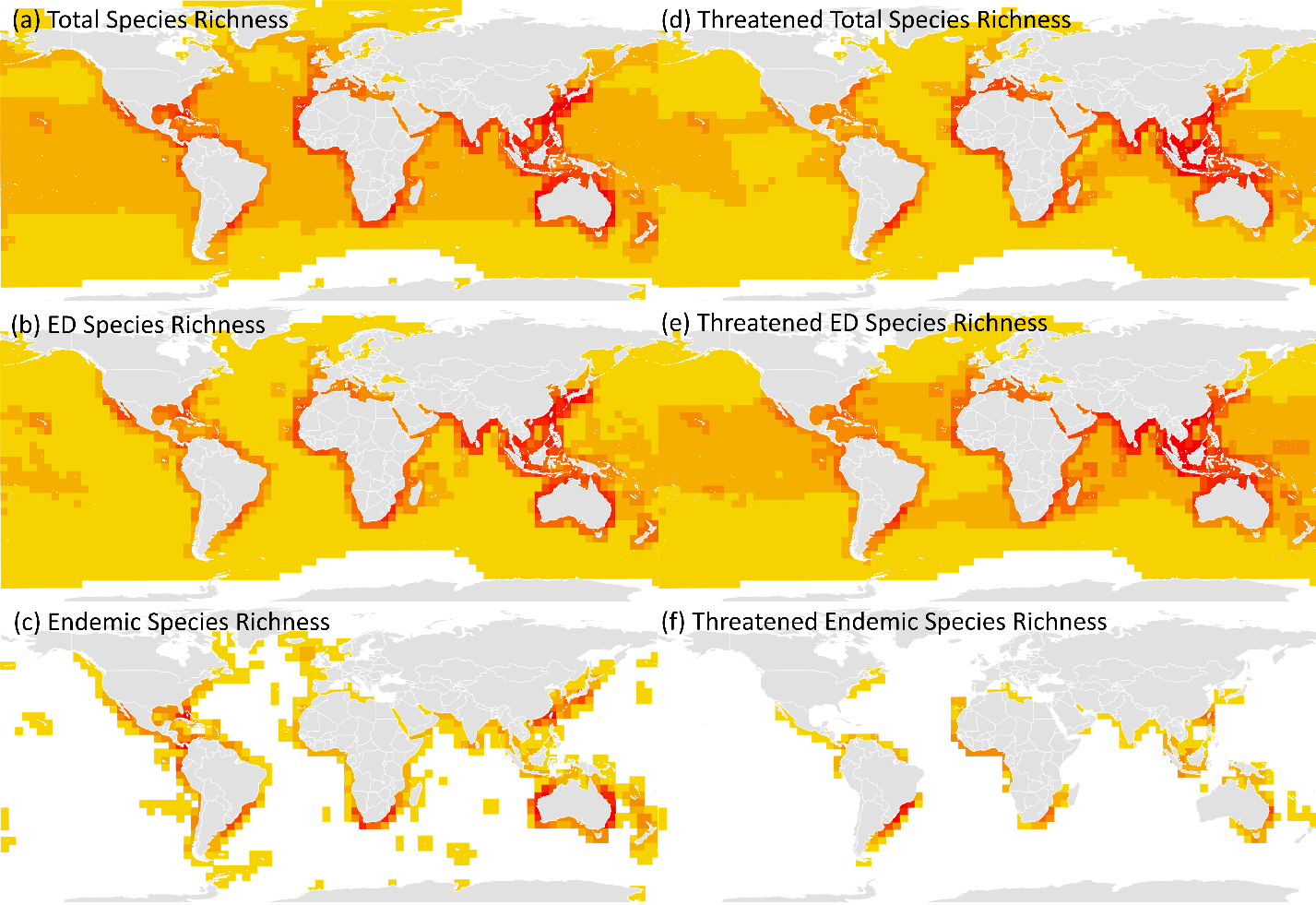
**

**S1 Fig. Global biodiversity patterns for three measures of species richness at 4° resolution.** General richness for (a) total species, (b) evolutionarily distinct (ED) species, and (c) endemic species. (d-f) Threatened subsets of richness patterns for (d) total species, (e) evolutionarily distinct (ED) species, and (f) endemic species. Geographic coordinate system is in NAD83, projected coordinate system is lambert equal area. The data used for this figure under CC BY license is granted permission from the International Union for the Conservation of Nature (IUCN), original copyright 2011.
