# Supplementary material for: Spatially congruent sites of importance for global shark and ray biodiversity
Source: PLoS One. 2020 Jul 6;15(7):e0235559. doi: 10.1371/journal.pone.0235559 (PMC7337351; doi:10.1371/journal.pone.0235559)
Supplement: S3 Fig — Congruency is represented at two levels of hotspot definition: (a) 2.5% and (b) 10%, and three levels of spatial resolution: 1°, 4°, and 8°. The subset of threatened species only are indicated in red. (DOCX) [file pone.0235559.s003.docx]

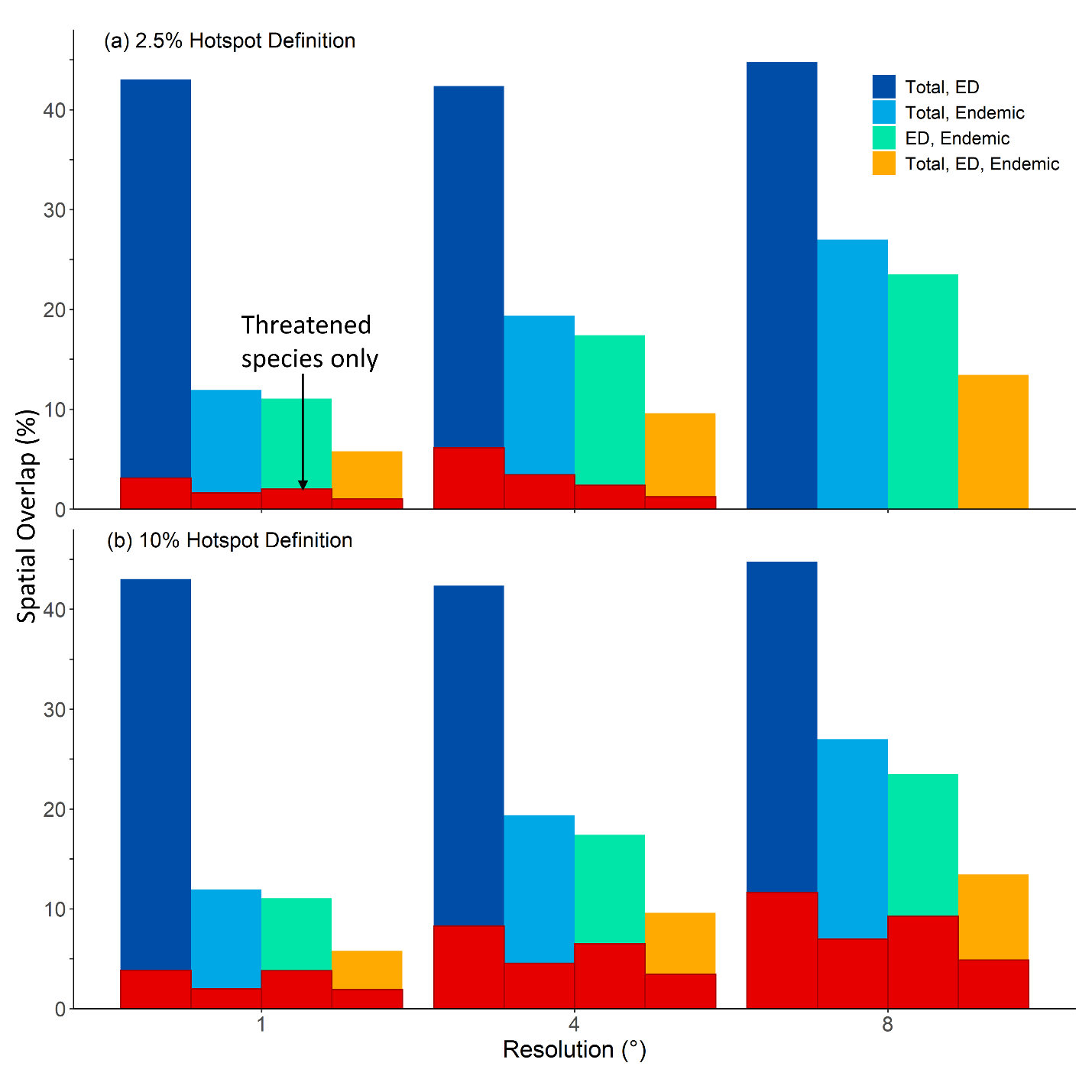


**S3 Fig. Spatial congruency (measured as percent overlap) of shark hotspots between three species richness measures: total species, evolutionary distinct (ED) species, and endemic species.** Congruency is represented at two levels of hotspot definition: (a) 2.5% and (b) 10%, and three levels of spatial resolution: 1°, 4°, and 8°, for total number of species and the subset of threatened species indicated in red.
