# Supplementary material for: Spatially congruent sites of importance for global shark and ray biodiversity
Source: PLoS One. 2020 Jul 6;15(7):e0235559. doi: 10.1371/journal.pone.0235559 (PMC7337351; doi:10.1371/journal.pone.0235559)
Supplement: S7 Fig — General richness hotspots of (a) total species, (b) evolutionarily distinct (ED) species, and (c) endemic species. (d-f) Threatened subset of richness hotspots for (d) total species, (e) evolutionarily distinct (ED) species, and (f) endemic species. For each species richness measures, hotspots are defined as the richest 5% of grid cells. Geographic coordinate system is in NAD83, projected coordinate system is lambert equal area, grid cell resolution is 4°. The data used for this figure under CC BY license is granted permission from the International Union for the Conservation of Nature (IUCN), original copyright 2011. (DOCX) [file pone.0235559.s007.docx]

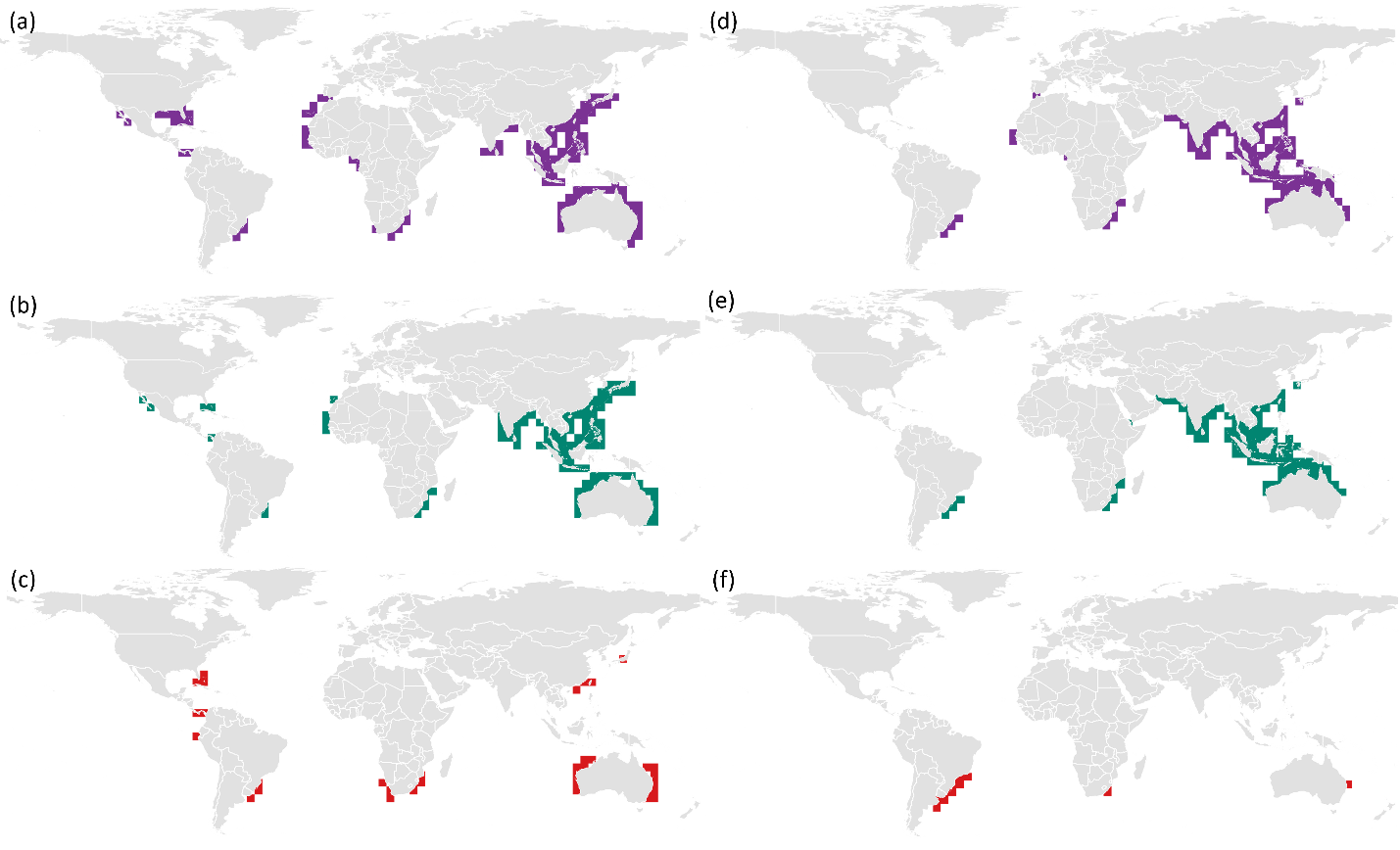


**S7 Fig. Biodiversity hotspots derived for three measures of species richness.** General richness hotspots of (a) total species, (b) evolutionarily distinct (ED) species, and (c) endemic species. (d-f) Threatened subset of richness hotspots for (d) total species, (e) evolutionarily distinct (ED) species, and (f) endemic species. For each species richness measures, hotspots are defined as the richest 5% of grid all cells. Geographic coordinate system is in NAD83, projected coordinate system is lambert equal area, grid cell resolution is 4°. The data used for this figure under CC BY license is granted permission from the International Union for the Conservation of Nature (IUCN), original copyright 2011.
