# Supplementary material for: Spatially congruent sites of importance for global shark and ray biodiversity
Source: PLoS One. 2020 Jul 6;15(7):e0235559. doi: 10.1371/journal.pone.0235559 (PMC7337351; doi:10.1371/journal.pone.0235559)
Supplement: S13 Fig — Spatially congruent areas between total species, ED species, and endemic species at (a) 1° resolution, (b) 4° resolution, and (c) 8° resolution, and (d-f) for the subset of threatened species, corresponding to resolution levels of (a-c). The data used for this figure under CC BY license is granted permission from the International Union for the Conservation of Nature (IUCN), original copyright 2011. (DOCX) [file pone.0235559.s013.docx]

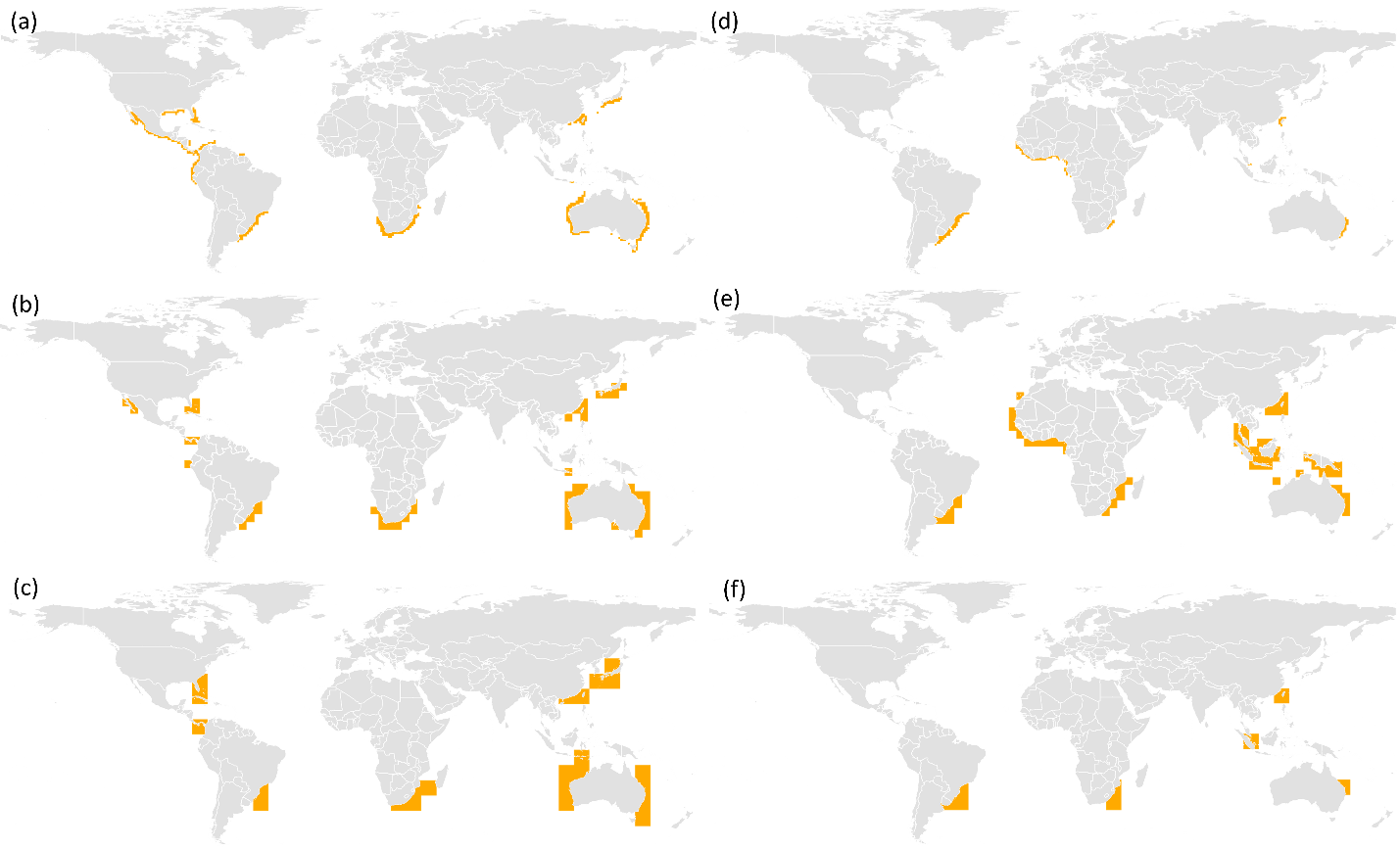


**S13 Fig. Spatially congruent areas between biodiversity hotspots derived from different species richness measures represented as the richest 10% of grid all cells.** Spatially congruent areas between total species, ED species, and endemic species at (a) 1°resolution, (b) 4° resolution, and (c) 8° resolution, and (d-f) for the subset of threatened species, corresponding to resolution levels of (a-c). The data used for this figure under CC BY license is granted permission from the International Union for the Conservation of Nature (IUCN), original copyright 2011.
