# Supplementary material for: Spatially congruent sites of importance for global shark and ray biodiversity
Source: PLoS One. 2020 Jul 6;15(7):e0235559. doi: 10.1371/journal.pone.0235559 (PMC7337351; doi:10.1371/journal.pone.0235559)
Supplement: S1 Table — Congruency is compared between three levels of spatial resolution: 1°, 4°, and 8°, for total number of species and the subset of threatened species, and at three levels of defining hotspot (2.5%, 5%, and 10% of richest cells). (DOCX) [file pone.0235559.s014.docx]

**S1 Table. Spatial congruency (measured as percent overlap) of shark hotspots between three species richness metrics: total species, evolutionary distinct (ED) species, and endemic species.** Congruency is compared between three levels of spatial resolution: 1°, 4°, and 8°, for total number of species and the subset of threatened species, and at three levels of defining hotspot (2.5%, 5%, and 10% richest cells).

|  |  |  | **Spatial congruency between richness measures (% overlap)** | | | |
| --- | --- | --- | --- | --- | --- | --- |
| **Cell size** | **Percent Richest cells (%)** | **Species category** | **Total \|  ED** | **Total \| Endemic** | **Endemic \| ED** | **Total \| Endemic \| ED** |
| **1°** | 2.5 | total | 41.42 | 11.89 | 10.77 | 5.63 |
|  |  | threatened | 3.12 | 1.62 | 2.02 | 1.04 |
|  | 5 | total | 43.03 | 11.93 | 11.07 | 5.78 |
|  |  | threatened | 4.02 | 2.11 | 3.05 | 1.51 |
|  | 10 | total | 45.68 | 12.47 | 12.26 | 6.38 |
|  |  | threatened | 3.83 | 2.00 | 3.81 | 1.93 |
| **4**° | 2.5 | total | 37.81 | 21.94 | 15.76 | 8.61 |
|  |  | threatened | 6.14 | 3.44 | 2.41 | 1.24 |
|  | 5 | total | 42.35 | 19.37 | 17.41 | 9.60 |
|  |  | threatened | 4.76 | 2.64 | 4.24 | 2.15 |
|  | 10 | total | 45.35 | 19.11 | 18.46 | 10.11 |
|  |  | threatened | 8.28 | 4.55 | 6.54 | 3.43 |
| **8**° | 2.5 | total | 36.41 | 29.71 | 21.39 | 12.34 |
|  |  | threatened | 0 | 0 | 0 | 0 |
|  | 5 | total | 44.76 | 26.97 | 23.49 | 13.43 |
|  |  | threatened | 6.27 | 3.76 | 4.81 | 2.50 |
|  | 10 | total | 44.39 | 26.42 | 25.74 | 14.58 |
|  |  | threatened | 11.64 | 6.98 | 9.24 | 4.87 |
